# The Prognostic Accuracy Evaluation of mNUTRIC, APACHE II, SOFA, and SAPS 2 Scores for Mortality Prediction in Patients with Sepsis

**DOI:** 10.1155/2022/4666594

**Published:** 2022-10-13

**Authors:** Pham Dang Hai, Le Thi Viet Hoa

**Affiliations:** ^**1**^ Medical Intensive Care Unit, 108 Military Central Hospital, Ha Noi, Vietnam; ^2^Intensive Care Unit, Tam Anh General Hospital, Ha Noi, Vietnam

## Abstract

**Background:**

The modified Nutrition Risk in the Critically Ill (mNUTRIC) score is a helpful tool to evaluate nutritional risk in critically ill patients. However, there is a lack of data on the relationship between mNUTRIC score and septic patients' outcomes. So, this study aims to validate the prognostic role of the mNUTRIC score and to compare the performances of mNUTRIC, APACHE II, SOFA, and SAPS 2 scores for mortality prediction in patients with sepsis.

**Methods:**

This prospective observational study was performed on 194 septic patients admitted to the Intensive Care Unit (ICU) of 108 Military Central Hospital. Sepsis was defined based on the sepsis-3 definition. The mNUTRIC score was used to evaluate the nutritional status within 24 h of ICU admission. Baseline characteristics and clinical information were collected to calculate the mNUTRIC, APACHE II, SOFA, and SAPS 2 scores. The outcome was in-hospital mortality from all causes.

**Results:**

Nonsurvivors patients had a significantly higher median mNUTRIC score (6 vs. 4, *P* < 0.001). The mortality rate in the group with a NUTRIC score ≥5 was significantly higher than in the group with a NUTRIC score <5 (56.0% vs 10.2%; *P* < 0.001). The area under the ROC curves (AUC) for predicting the mortality of mNUTRIC was 0.79 (sensitivity 67.1% and specificity 81.0% (*P* < 0.001)). Compared with other severity scores in mortality prediction, AUC was 0.78 for APACHE II (sensitivity 84.9% and specificity 67.7%), 0.77 for SOFA score (sensitivity 76.7% and specificity 65.3%), and 0.73 for SAPS 2 (sensitivity 66.1%, specificity 77.7%). In the multivariate analysis, mNUTRIC score was associated with in-hospital mortality (HR, 2.00; 95% CI, 1.54 to 2.58; *P* < 0.001).

**Conclusions:**

Our study showed that the mNUTRIC score was similar to severity scores (APACHE II, SOFA, SAPS 2) in mortality prediction and was the independent mortality predictor in patients with sepsis.

## 1. Introduction

Sepsis is a common mortality cause in critically ill patients, with a high in-hospital mortality of around 25–30% [1–3]. Sepsis is characterized by the characteristics of a robust inflammatory response combined with an acute catabolic state that leads to the breakdown of glycogen, lipid, and protein stores to drive glucose production [4]. Several risk factors are associated with elevated mortality rates in sepsis and septic shock, including advanced age, impaired host immune function, the severity of illness, treatment strategies, and malnutrition [5–8].

Approximately 30% to 69% of patients presenting to the intensive care unit (ICU) are malnourished [9–11]. Many patients were malnourished upon hospital admission or became malnourished during hospitalization [10, 12]. Patients with preexisting malnutrition are associated with organ dysfunction, impaired immune function, delayed wound healing, rising health care costs, prolonged hospital stays, and increased mortality risk [13–15]. An adequate nutritional risk assessment has essential value for ICU patients. Under supportive and appropriate nutritional therapy, hospital undernutrition can be decreased and improve outcomes [16].

Although several tools for nutritional risk screening, including the Subjective Global Assessment and Nutritional Risk Screening 2002, have been applied, they are unsuitable for ICU patients [16, 17]. The Nutrition Risk in the Critically Ill Score, using nutritional risk assessment, was first reported in 2011 for ICU patients. This score has six components: age, APACHE II score, SOFA score, serum interleukin 6 (IL-6) concentration, number of comorbidities, and number of days in hospital until admission to the ICU [18]. However, IL-6, a marker of inflammation, is not routinely performed in the ICU. Therefore, a modified version of the NUTRIC score (mNUTRIC) without IL-6 was proposed in 2016 [19]. However, data are limited regarding the relationship between the mNUTRIC score and mortality in septic patients, especially Asian patients.

The study aimed to validate the predictive value of the mNUTRIC score in septic patients and compare the performance of mNUTRIC, APACHE II, SOFA, and SAPS 2 scores for mortality prediction in patients with sepsis. In addition, we hypothesized that an elevated mNUTRIC score is associated with mortality in septic patients.

## 2. Materials and Methods

### 2.1. Patients

A prospective cross-sectional study was performed in the ICU between December 2016 and December 2018. All patients over 18 years old with sepsis, according to the sepsis-3 definition [20], were recruited for this study. Sepsis was confirmed as a SOFA score of 2 points or more from the baseline consequent to the infection [20]. Septic shock was identified as sepsis associated with hypotension requiring vasopressors to maintain a mean arterial pressure of 65 mmHg or greater and a blood lactate concentration of more than two mmol/L despite adequate fluid resuscitation [20]. Exclusion criteria included patients who died or were discharged within the first 24 h of ICU admission and patients or their family members who refused participation.

The Institutional Review Board of our hospital approved the protocol for the research. Informed consent was collected from the patients or their family members.

### 2.2. Data Collection

The patient data that were collected, including demographic variables (age, sex), medical history, vital signs, length of ICU stay, mechanical ventilation, the dosage of noradrenaline, results of blood culture, laboratory parameters (white blood cells, neutrophils, platelets, creatinine, procalcitonin, lactate, liver enzymes, total bilirubin), and the clinical outcomes (in-hospital mortality).

The Acute Physiology and Chronic Health Evaluation II (APACHE II) score [21], the Sequential Organ Failure Assessment (SOFA) [22], and the Simplified Acute Physiology Score (SAPS) 2 [23] were measured within 24 h after ICU admission.

The mNUTRIC score (0–9 points) was calculated from data collected 24 h after ICU admission. The mNUTRIC score included five variables: age, number of comorbidities, SOFA score, APACHE II score, and days of hospitalization before ICU admission [19].

### 2.3. Statistical Analysis

All analyses were performed using SPSS version 20.0 and Epi info 2005 (version 3.3.2) for the window. Categorical variables were described as frequencies (percentages). Continuous variables were introduced as mean values ± standard deviation (SD) for parametric variables or median (interquartile range) for nonparametric variables. Categorical variables were analyzed according to the chi-square test or the Fisher's exact test, as appropriate. The student's *t*-test was introduced for normally distributed quantitative data, and the Mann–Whitney test was introduced for non-normally distributed data.

The value of the mNUTRIC score for predicting mortality was evaluated by the AUC of the receiver operating characteristic (ROC) curve to detect the cutoff value of the mNUTRIC score for predicting in-hospital mortality in septic patients. The best cutoff point was selected as the maximum value of the sum of sensitivity and specificity [24].

Univariate analyses were used to confirm mortality-related factors. Multivariate logistic regressions were used to assess independent predictors of mortality. Associations of parameters with the risk of death were expressed as hazard ratio (HR). The *P* value was considered statistically significant when it was less than 0.05.

## 3. Results

### 3.1. Baseline Characteristics of Patients

One hundred ninety-four patients with sepsis, 143 (73.7%) males and 51 (26.3%) females, were included. The baseline features of the study subjects are introduced in Table 1.

The median septic patient age was 69 years (IQR: 59–80 years), with a median APACHE II score of 18, a median SOFA score of 10, and a median SAPS 2 score of 44.

Most patients had a medical history, including 49 patients (25.3%) with diabetes, 28 patients (14.4%) with stroke, and 78 patients (40.2%) with hypertension. One hundred forty-one patients (72.6%) had septic shock, and the remaining 53 (27.4%) had sepsis. One hundred sixty-two patients (83.5%) received mechanical ventilation. The median duration of patients' stays in the ICU was five days (IQR: 3–9 days). The in-hospital death rate was 37.6%.

**Figure 1:**
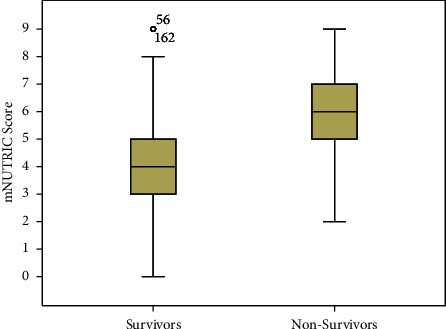
mNUTRIC score in survivors and nonsurvivors groups. mNUTRIC score: median 4 vs 6, *P* < 0.001.

The age, sex, and ICU length of stay in the two groups showed no significant difference (*P* > 0.05). The percentage of mechanical ventilation, septic shock, heart rate, and severity of illness scores, including SOFA score, APACHE II score, and SAPS 2 score in nonsurvivors, were significantly higher than in survivors (*P* < 0.001). The modified NUTRIC score in nonsurvivors was significantly higher compared to survivors (median (IQR) 6 (5–7) versus 4 (3–5), *P* < 0.001) (Table 1 and Figure 1).

Nonsurvivors had significantly lower platelets (*P*=0.012). Lactate levels and proportion of bacteremia were significantly higher in nonsurvivors than in survivors. There was no significant difference in neutrophils, hemoglobin, procalcitonin, creatinine, bilirubin, total liver enzymes, or glucose between the two groups (*P* > 0.05) (Table 2).

### 3.2. Prognostic Accuracy of mNUTRIC, APACHE II, SOFA, and SAPS 2 Scores in Septic Patients

**Figure 2:**
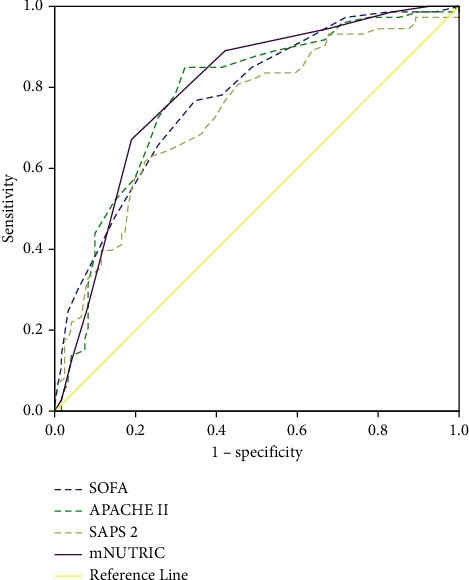
Performance of different scoring systems to predict in-hospital mortality in septic patients.

The result of the ROC analysis to detect mortality were presented in Figure 2.

**Figure 3:**
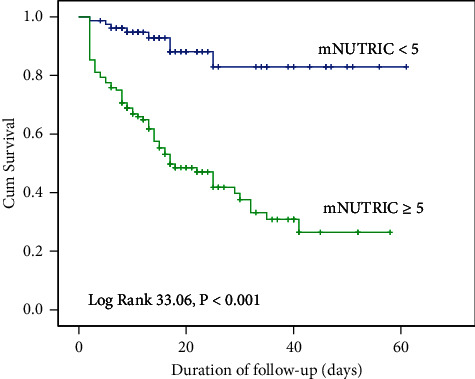
Mortality of septic patients in-hospital according to mNUTRIC score < 5 or mNUTRIC score ≥ 5.

The AUC of the mNUTRIC score for predicting in-hospital mortality was 0.79 (95% CL: 0.73–0.85), and the optimal cutoff was 5 (sensitivity 67.1% and specificity 81.0%). When we compared different severity scores with mortality, the cutoff points were 17 for APACHE II (sensitivity 84.9% and specificity 67.7%, AUC = 0.78), 9 for SOFA (sensitivity 76.7% and specificity 65.3%, AUC = 0.77), and 48 for SAPS 2 (sensitivity 66.1%, specificity 77.7%, AUC = 0.73). The detailed results are shown in Table 3. A group of NUTRIC score ≥5 had a significantly higher mortality rate than a group of mNUTRIC score <5 (56.0% vs 10.2%; *P* < 0.001) (Figure 3).

### 3.3. Modified NUTRIC Score with Mortality

In univariate Cox proportional hazards models (Table 4), mNUTRIC score, SOFA, APACHE II, SAPS 2 scores, and septic shock were related to in-hospital mortality (*P* < 0.001). The Cox proportional hazard regression analysis showed that mNUTRIC score (HR, 2.00; 95% CI, 1.54 to 2.58; *P* < 0.001) was the independent predictor for in-hospital mortality.

## 4. Discussion

Malnutrition is common in ICU patients and often undetected and untreated due to inadequate nutritional knowledge of hospital staff [25, 26]. Therefore, nutritional risk screening plays an important role and is the first step in interventional nutrition guidance [27]. According to ESPEN guidelines, a nutritional screening assessment aims to identify the likelihood of a better or worse outcome depending on nutritional status [25]. An adequate nutritional regimen is believed to reduce the metabolic response to stress, preclude cell damage from oxidative stress, regulate the immune system's reaction, and lead to a reduction in the severity of the disease, reduced complications, decreased length of ICU stay, and improved outcomes in ICU patients [28, 29].

The NUTRIC score was the first appropriate nutritional risk screening tool for ICU patients. It was developed by Heyland et al. [18]. Later, Rahman proposed the modified NUTRIC score by excluding the serum IL-6 concentration, which is rarely measured in clinical practice [19]. mNUTRIC is a promising nutritional risk assessment and screening tool for critically ill patients [30].

The main study finding showed that the mNUTRIC score was significantly higher in nonsurvivors with sepsis and was associated with in-hospital mortality. The mortality in the group of mNUTRIC ≥5 was significantly higher than in the group of mNUTRIC <5 (56.0% vs. 10.2%; *P* < 0.0001). The results were similar to those of other studies [8, 31]. Jeong DH et al. demonstrated that septic patients with the increased mNUTRIC score had higher 28‐day mortality [8]. Mukhopadhyay et al. demonstrated that the mNUTRIC score in nonsurvivors was higher than in survivors (6.16 vs. 4.67, *P* < 0.001) in 401 intensive care patients [32]. A meta-analysis conducted from 8 studies with 4076 critically ill patients by Ibrahim DA et al. reported that a high mNUTRIC score (≥5) was associated with an increased risk of 28-day mortality (RR 2.025; 95% CI 1.488–2.758; *P* < 0.001) and an increased length of stay in ICU (95% CI 1.78–4.99 days; *P* < 0.001) [30].

Sepsis may exacerbate malnutritional status by a severe catabolic response during the acute phase of the disease, combined with an exacerbated proinflammatory state, reduced gastrointestinal motility, poor absorption, and prolonged immobility, leading to muscle wasting and hospital-acquired infections [33]. The physiological synergism between malnutrition and infection has been recognized [34]. Malnutrition is one of the independent factors associated with an increased mortality rate and risk of adverse events [35]. Malnutrition affects poor outcomes via several mechanisms. Poor nutrition causes a change in systemic regulatory functions and immune system deficiency [33]. Moreover, malnutrition is associated with increased intestinal permeability, resulting in increased translocation of intestinal flora, especially bacteria, across the intestinal epithelial barrier [36]. Besides, hypoalbuminemia may reduce the efficiency of transportation of highly protein-bound antibiotics and increase the rate of drug filtration and elimination [37].

The mNUTRIC score is a rapid assessment of nutritional status based on illness severity, including SOFA, APACHE II score, age, and comorbidities [19]. However, the best mNUTRIC score cutoff point for predicting mortality in patients with sepsis is still uncertain. The results of our study showed that the area under the curve of mNUTRIC for in-hospital mortality prediction was 0.79 with the best cutoff of 5 (specificity 81.0% and sensitivity 67.1%) and was similar to the AUCs of the APACHE II score, SOFA score, and SAPS 2 score. These findings agreed with Mukhopadhya; an AUC for 28-day mortality in critically ill patients was 0.71, with the best cutoff found at 5 (sensitivity of 72% and specificity of 63%) [32]. In another study, Shukeri W studied 432 critically ill patients in Malaysia and showed that the mNUTRIC score had good predictive performance with an AUC of 0.79 and an optimal cutoff of 6 [31].

In contrast to a previous study, Jeong DH et al. reported that the best cutoff of mNUTRIC for predicting mortality in sepsis was 6 (sensitivity 75.3% and specificity 64.8%) [8]. This difference may be related to different study populations, sample sizes, treatment interventions, and therapy. Further studies are needed to identify the optimal cutoff value of the mNUTRIC for the high-risk group, especially in sepsis and septic shock.

When we compared different severity scores with mortality, the predictive value of mNUTRIC was similar to APACHE II, SOFA, and SAPS 2. These findings of our study are similar to the results of Kumar et al. The authors showed that the mNUTRIC score was similar to the APACHE II and SOFA scores for mortality prediction in critically ill patients [38].

### 4.1. Limitations

Our research has some limitations. First, the research was performed at a single center with a small sample size and an Asian population, so its findings cannot be generalized to the larger external population. Larger multicenter studies should be considered to confirm our findings. Second, dynamic nutritional risk assessments have not been evaluated, which can supply additional information on patient outcomes. Furthermore, the impact of nutritional status on the death rate in individuals with different mNUTRIC scores has not been analyzed.

## 5. Conclusion

Our study demonstrated that the mNUTRIC score was similar to other severity scores (APACHE II, SOFA, SAPS 2) in mortality prediction and was the independent predictor in septic patients. The mNUTRIC score might be a valuable tool for predicting the prognosis of septic patients. However, further studies need to be done to confirm our findings.

## Figures and Tables

**Table 1:** Baseline clinical characteristics of patients.

	Total (n = 194)	Survivors (*n* = 121)	Nonsurvivors (*n* = 73)	*P* Value
*Characteristics*				
Age, years	69 (59–80)	69 (59–78)	69 (57–83)	0.630
Male sex, *n* (%)	143 (73.7)	87 (71.9)	56 (76.7)	0.463
Septic shock, *n* (%)	141 (72.6)	79 (65.3)	62 (84.9)	0.002^*∗*^
Noradrenaline dose, *μ*g/kg/min	0.18 (0.00–0.40)	0.10 (0.00–0.30)	0.25 (0.10–0.50)	0.477
Mechanical ventilation, *n* (%)	162 (83.5)	91 (75.2)	71 (95.9)	0.001^*∗*^
Length of ICU stay, days	5 (3–9)	5 (3–9)	5 (2–10)	0.724

*Comorbidities*				
Hypertension, *n* (%)	78 (40.2)	47 (38.8)	31 (42.7)	0.620
Diabetes mellitus, *n* (%)	49 (25.3)	24 (19.8)	25 (34.2)	0.025
Stroke, *n* (%)	28 (14.4)	18 (14.8)	10 (13.7)	0.822
COPD, *n* (%)	10 (5.2)	3 (2.5)	7 (9.6)	0.061

*Source of infection*				
Abdominal	86 (44.3)	68 (56.2)	18 (24.6)	
Respiratory	82 (42.3)	42 (34.7)	40 (54.8)	
Urinary tract	10 (5.2)	6 (4.9)	4 (5.5)	
Skin	11 (5.7)	1 (0.8)	10 (13.7)	
Other	5 (2.6)	4 (3.4)	1 (1.4)	

*Initial physiologic variables,*				
Heart rate, bpm	107 (90–120)	100 (90–120)	110 (100–120)	0.008
MAP, mmHg	73.0 (63.0–85.0)	75.5 (70.0–88.0)	70.0 (60.0–73.0)	0.001^*∗*^

*Severity of illness*				
SOFA score	10 (6–12)	7 (5–11)	11 (10–14)	<0.001^*∗*^
APACHE II score	18 (13–23)	14 (11–20)	23 (19–27)	<0.001^*∗*^
SAPS 2 score	44 (34–56)	39 (39–47)	53 (42–61)	<0.001^*∗*^
mNUTRIC score	5 (4–6)	4 (3–5)	6 (5–7)	<0.001^*∗*^
mNUTRIC ≥5	116 (59.8)	51 (42.1)	65 (89.0)	<0.001^*∗*^

Data are introduced as median(interquartilerange) and number (*n*) of patients (%), as appropriate. COPD, chronic obstructive pulmonary disease; APACHE II, acute physiology and chronic health evaluation; ICU, intensive care unit; MAP, mean arterial pressure; mNUTRIC, modified Nutrition Risk in Critically Ill; SOFA, sequential organ failure assessment; SAPS 2, Simplified Acute Physiology Score 2; ^*∗*^*P* < 0.05.

**Table 2:** Baseline laboratory data at admission.

	Total (*n* = 194)	Survivors (*n* = 121)	Nonsurvivors (*n* = 73)	*P* Value
WBC, ×10^9^/L	12.4 (7.2–21.6)	13.2 (7.4–23.0)	11.8 (6.0–17.2)	0.051
Neutrophils, *n* (%)	87.3 (80.9–92.4)	88.7 (81.4–92.8)	86.8 (80.3–91.9)	0.437
Hemoglobin, g/L	109 (96–130)	111 (97–131)	106 (94–126)	0.496
Platelet, ×10^9^/L	176 (99–262)	193 (121–286)	143 (67–207)	0.012^*∗*^
Procalcitonin, ng/mL	35.5 (8.0–100.0)	35.2 (6.5–100.0)	35.5 (13.8–100.0)	0.885
Lactate, mmol/L	3.8 (2.3–7.2)	3.3 (2.0–6.4)	4.4 (2.7–8.3)	0.025
Creatinine, µmol/L	149 (98–250)	143 (88–226)	173 (104–271)	0.272
Bilirubin total, µmol/L	17.9 (11.1–39.9)	17.7 (10.9–40.9)	18.5 (11.5–36.0)	0.902
AST, u/l	74 (37–152)	69 (33–150)	83 (42–195)	0.233
ALT, u/l	41 (20–90)	40 (19–90)	45 (21–89)	0.633
Glucose, mmol/L	7.5 (5.7–11.2)	7.5 (5.7–11.2)	7.8 (5.8–11.2)	0.646
*Bacteremia*	62 (32.0)	32 (26.4)	30 (41.1)	0.034

Data are introduced as median (interquartile range). AST, aspartate transaminase; ALT, alanine aminotransferase; WBC, white blood cell; ^*∗*^*P* < 0.05.

**Table 3:** The performance of different variables for predicting mortality.

Variables	AUC	Value	Specificity (%)	Sensitivity (%)	*P*
APACHE II	0.78	17	67.7	84.9	<0.001
SOFA	0.77	9	65.3	76.7	<0.001
SAPS 2	0.73	48	77.7	61.6	<0.001
mNUTRIC	0.79	5	81.0	67.1	<0.001

AUC, area under the curve; mNUTRIC, modified Nutritional Risk in Critically Ill; SAPS 2, Simplified Acute Physiology Score 2; APACHE II, acute physiology and chronic health evaluation; SOFA, sequential organ failure assessment.

**Table 4:** Univariate and multivariate analysis of in-hospital mortality in sepsis.

	Univariable		Multivariable	
Variables	HR (95% CI)	P	HR (95% CI)	P
Age	1.00 (0.98–1.02)	0.63	—	—
Male sex	1.28 (0.65–2.52)	0.46	—	—
Septic shock	2.99 (1.42–6.29)	0.004	1.01 (0.42–2.41)	0.983
APACHE II	1.14 (1.09–1.20)	<0.001	—	—
SAPS 2	1.06 (1.04–1.09)	<0.001	—	—
SOFA score	1.33 (1.20–1.46)	<0.001	—	—
mNUTRIC	1.99 (1.57–2.53)	<0.001	2.00 (1.54–2.58)	<0.001
mNUTRIC ≥5	11.15 (4.92–25.27)	<0.001	—	—

*C*I, confidence interval; mNUTRIC, modified Nutritional Risk in Critically Ill; APACHE II, acute physiology and chronic health evaluation; SOFA, sequential organ failure assessment; SAPS 2, Simplified Acute Physiology Score 2.

## Data Availability

The data used for the findings of this study are available from the corresponding author upon request.

## Abbreviations

ALT: Alanine aminotransferase
AST: Aspartate transaminase
APACHE II: Acute physiology and chronic health evaluation
DM: Diabetes mellitus
Hb: Hemoglobin
HR: Heart rate
CI: Confidence interval
COPD: Chronic obstructive pulmonary disease
LOS: Length of stay
MAP: Mean arterial pressure
mNUTRIC: Modified Nutrition Risk in Critically Ill
SAPS: Simplified Acute Physiology Score
SOFA: Sequential organ failure assessment
PCT: Procalcitonin
WBC: White blood cell.
